# Investigation on the Basic Characteristics of Semi-Fixed Abrasive Grains Polishing Technique for Polishing Sapphire (α-Al_2_O_3_)

**DOI:** 10.3390/ma15113995

**Published:** 2022-06-03

**Authors:** Yang Lei, Ming Feng, Ke Wu, Jinxi Chen, Jianghao Ji, Julong Yuan

**Affiliations:** 1 Special Equipment Institute, Hangzhou Vocational & Technical College, Hangzhou 310018, China; leiyangcn@126.com (Y.L.); hzgky2021@126.com (J.C.); jijianghao2020@126.com (J.J.); 2 College of Mechanical and Electrical Engineering, Wenzhou University, Wenzhou 325035, China; 3 Faculty of Mechanical Engineering & Mechanics, Ningbo University, Ningbo 315000, China; wkhz2020@126.com; 4 Ultra-Precision Machining Center, Zhejiang University of Technology, Hangzhou 310018, China; yjlhz2022@126.com

**Keywords:** sapphire, solid-phase reaction, polishing

## Abstract

Single-crystal sapphire (α-Al_2_O_3_) is an important material and widely used in many advanced fields. The semi-fixed abrasive grain processing method based on solid-phase reaction theory is a prominent processing method for achieving ultra-precision damage-free surfaces. In order to develop the proposed method for polishing sapphire, the basic characteristics of the semi-fixed abrasive grains polishing tool for polishing sapphire were determined. Weight analysis was used to study the influence rules of parameters on surface roughness and material removal rates using an orthogonal experiment. Then, the optimized polishing tool was obtained through a mixture of abrasive particle sizes to reduce the difficulty in molding the polishing tool. Finally, polishing experiments using different polishing tools were carried out to investigate polishing performance by considering the surface roughness, material removal rate and the surface morphology during polishing. The results showed that (1) external load affects the surface roughness and material removal rate the most, followed by abrasive particle size, sand bond ratio, revolution speed of the workpiece and he polishing tool; (2) the difficulty in manufacturing the polishing tool could be reduced by mixing larger abrasive particles with small abrasive particles; (3) the polishing tool with 200 nm and 1 μm particle sizes performed best in the first 210 min polishing.

## 1. Introduction

Single-crystal sapphire (α-Al_2_O_3_) has been widely used in many advanced situations, such as for use as semiconductor products, sensors, substrates and screen for smart devices, owing to its excellent physical and chemical properties, including high hardness, excellent thermal conductivity and capabilities in wear and corrosion resistance [1,2]. According to the demands of optoelectronic and microelectromechanical systems, the processing efficiency and quality of single-crystal sapphire substrates should be improved to meet new requirements [3]. Polishing is the final process for manufacturing sapphire products and is essential for achieving a high surface roughness. It was well known that sapphire crystal is one of the typical hard–brittle and hard-cut materials and that traditional polishing techniques, such as mechanical polishing and conventional chemical mechanical polishing, have difficulty in obtaining a higher surface quality for sapphire work surfaces and the defects induced by these methods, such as chipping, scratches and sub-surface damage, should be decreased or eliminated [4,5,6]. In this case, is was critical to develop new polishing methods for attaining desirable processing efficiency and quality.

To date, fixed-abrasive polishing methods have been invented to further improve the polishing efficiency and surface quality of materials. However, these methods only yield obvious improvements in polishing efficiency and are not suitable for polishing curved surfaces [7]. Therefore, the flexible polishing concept was proposed, in which a soft polishing tool can change its form to adapt to the changes in a curved radius on a work surface. As a typical flexible polishing technique, the magnetorheological finishing (MRF) method has excellent performance for polishing and has been widely adopted in ultra-precision finishing for various materials [8,9], such as advanced ceramics, optical glass, metal alloys, etc. The viscosity and hardness of the formed semi-fixed abrasive ribbon under a magnetic field is controlled by the intensity and distribution of an external magnetic field [10]. Based on this method, several novel magnetic field-assisted polishing method have been developed, including magnetic compound fluid (MCF) polishing with a dynamic magnetic field and chemical-combined MRF polishing [11]. It has been found that an ultra-smooth surface without sub-surface damage can be easily attained using this method due to its flexible magnetic tool and good controllability. The material removal rates of these methods, however, have been shown to be undesirable for meeting production requirements. Based on the chemical mechanical polishing method, where a softened material is achieved on a base material surface and then removed using a soft abrasive, such as new PS/CeO_2_ composite abrasives with difference in shell thicknesses, proposed by Chen [12]. The polishing results demonstrated that this kind of abrasive improved surface roughnesses because of the soft shell compared with those polished using free CeO_2_ abrasives. Gutsthe [13] polished sapphire substrates using colloidal SiO_2_ abrasives and achieved a removal efficiency of 0.4 μm/min. Further, in order to reduce the damage by CMP slurry to the surroundings, a novel green CMP slurry, where nano-sized silica particles were blended into deionized water with triethanolamine and sodium metasilicate nonahydrate, was invented by Zhang [14]. An ultra-smooth surface with an *R*_a_ of 0.11 nm was obtained, while a material removal rate of 3.31 μm/h was achieved. Gas–liquid-assisted CMP (GLA-CMP) was developed to reduce surface roughness. Compared with conventional CMP, the surface roughness (*R*_a_) of a sapphire wafer could be efficiently improved to 0.194 nm within 50 min using GLA-CMP [15].

However, these methods are all based on the traditional wet CMP method. Wang et al. conducted planarization processing on single-crystal *α*-alumina substrates and sapphire substrates using dry CMP with silica powder. It was found that the dry polishing method performs better than the wet polishing method, regardless of the surface quality or polishing efficiency [16]. Therefore, Yuan et al. proposed a semi-fixed abrasive grain processing method based on the solid-phase reaction theory to achieve an ultra-precision damage-free processing technique [17]. Wang et al. systematically studied the “trap” effect of semi-fixed abrasive grain processing [18] and clarified the definitions and categories of semi-fixed abrasive grain processing. Lev et al. studied the grinding mechanism of semi-fixed abrasives in the plastic domain and established a model for predicting the distribution of cutting depth using semi-fixed abrasives when processing silicon wafers and, finally, theoretically demonstrated the basic grinding characteristics of semi-fixed abrasive processing technology [19]. Based on the traditional Preston equation and the finite element method, a material-removal model of semi-fixed abrasive processing was proposed by Deng [20], where the influence on performance by semi-fixed abrasive and processing parameters was deeply investigated. In order to further develop the semi-fixed abrasive processing technique, Zhang et al. studied the lapping characteristics of sapphire using semi-fixed abrasive grain in the ductile domain, where the transformation characteristics of sapphire (from ductile to brittle) were analyzed and a mathematical model for the machining of sapphire in the ductile domain was established [21]. Zhang found that dry CMP, using semi-fixed abrasives based on a solid-phase reaction, was more efficient and precise in improving surface quality compared to using free abrasives on sapphire. In this case, Zhou et al. began comprehensive research on the semi-fixed soft abrasive polishing technique for sapphire wafers, based on a solid-phase reaction [22].

However, the current semi-fixed abrasive grains polishing technology for sapphire wafer only systematically studies the compositions and polishing failure performance of the semi-fixed soft abrasives and the basic characteristics for polishing sapphire wafers and optimization of polishing parameters were not investigated. Therefore, this paper investigates polishing sapphire based on semi-fixed abrasive grain processing technique for further developing its application in polishing fields.

## 2. Polishing Principle

**Figure 1:**
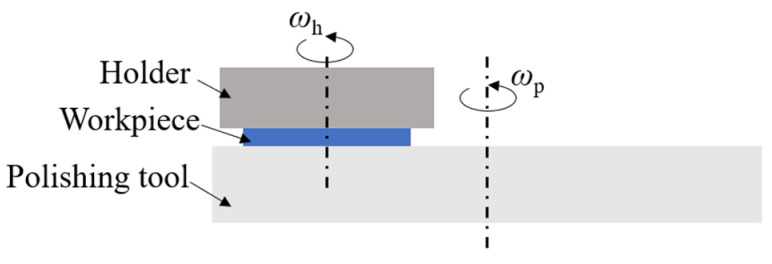
Diagram of polishing principle.

**Figure 2:**
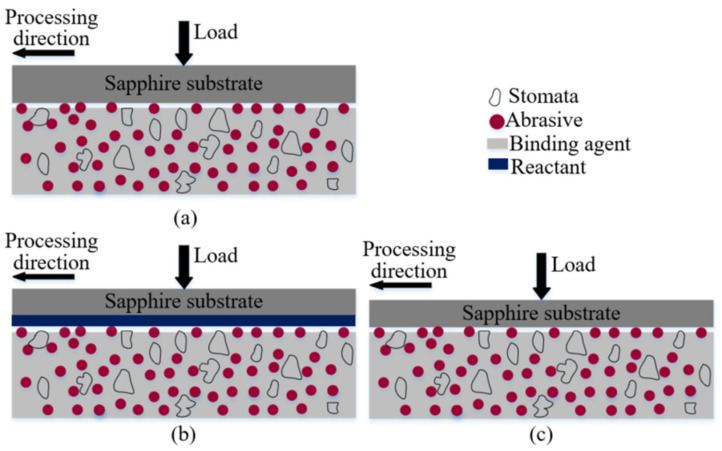
Diagram of polishing with semi-fixed soft abrasives: (**a**) Initial state of polishing (**b**) during polishing with reactant formation (**c**) during polishing with reactant removal.

Figure 1 shows the diagram of polishing principle. The workpiece was held by a holder which was also used for suffering external load when it was needed. The polishing tool was located beneath the workpiece. Once the polishing tool and holder were revolved on their axis, the workpiece can be polished. In order to clarify the polishing characteristics, the schematic diagram of polishing sapphire with semi-fixed soft abrasives was shown in Figure 2. As shown in Figure 2a, the polishing tool consists of abrasive particles, a binding agent and appropriate stomata to polish the workpiece under a certain pressure and relative movement speed. In the process of polishing, because the Moh’s hardness of soft abrasive particles is lower than that of sapphire, the sapphire surface will not be affected by soft abrasive particles. Abrasive particles react with sapphire substrates to obtain reactant, which has a lower hardness than abrasive particles (Figure 2b). After being mechanically removed by abrasive particles, reactant is continuously stripped from the substrate, and finally, the sapphire substrate is obtained without damaging the surface (Figure 2c). The distribution density of abrasive particles is far larger than that of traditional free abrasive particle processing in the contact area with sapphire; therefore, the processing efficiency is also higher. 

**Figure 3:**
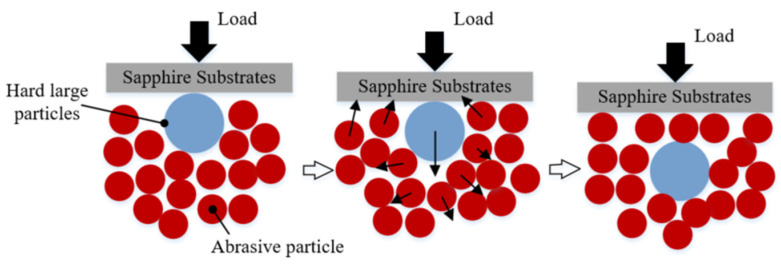
Schematic diagram of “trap” effect of semi-fixed abrasives during polishing.

At the same time, soft abrasive particles are in semi-fixed state, which means that free state of abrasive particles can be realized under certain conditions, leading to a “trap” effect during polishing. The “trap” effect will protect the substrate from being damaged by large and hard particles. The “trap” effect (schematic diagram shown in Figure 3) is expressed as: when the large and hard particles invade the sapphire processing process, the small-sized abrasive particles around the large and hard particle start to migrate in position, and then, the large and hard particle gradually sinks into the “trap” formed by the small-sized abrasive particles; finally, the machining load is still borne by the small-sized abrasive particles, thus avoiding the potential damage caused by the hard and large particles to the workpiece surface.

## 3. Processing Condition

### 3.1. Experimental Setup

**Figure 4:**
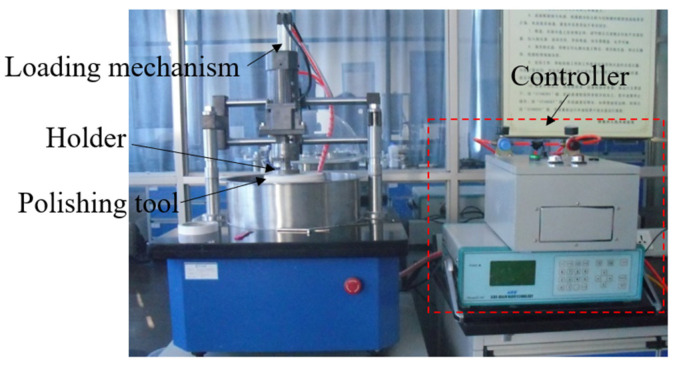
Experimental setup.

In order to carry out polishing experiments, based on the above-mentioned polishing principles, the corresponding experimental equipment was designed and manufactured, as shown in Figure 4. During processing, the sapphire was attached to the holder with paraffin wax by a placement machine. The holder was mounted to the motor to achieve independent rotation, which was installed on the loading mechanism to apply external loads. The polishing tool was fixed onto the built-in motor of the equipment to realize the rotation during processing. In addition, precise regulation of rotational speed and load can be implemented through the control system.

### 3.2. Experimental Conditions

The polishing performance of the semi-fixed abrasive particles was mainly determined by the abrasive particles and the binder. The main factors of the polishing process were the rotational speed of the workpiece and the polishing tool and external load. Therefore, 5 factors were used in this paper: (A) abrasive particle (SiO_2_) size, (B) mass ratio of abrasive grains to binder (hereafter called sand bond ratio, bonder: resin binder), (C) rotation speed of the workpiece, (D) rotation speed of the polishing tool and (E) external load and each factor has 3 levels. The number of factors was not higher than the number of columns in the orthogonal table. Without considering the interaction between the factors, the designed orthogonal table with 5 factors and 3 levels and two empty columns F and G was shown in Table 1. Each experiment was carried out for 120 min.

**Figure 5:**
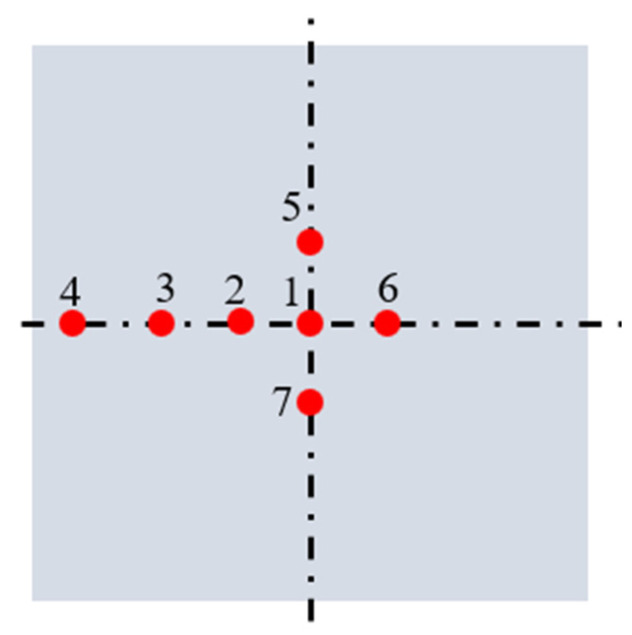
Selected points on the work surface.

Sapphire substrates with a crystal orientation of c<0001> were employed in the experiments (□40 × 0.5 in thickness mm). As shown in Figure 5, 7 points on the work surface (Figure 5) were selected for investigating the surface roughness *R*_a_ (mean value of each point) which was measured by a white light interferometer (Taylor Hobson CCI HD). Before measuring surface roughness, the substrate was immerged into dehydrated alcohol and cleaned by the ultrasonic cleaner for 30 min. After then, high pressure gas was used to dry the substrate. Then, the substrate was placed to the workbench of white light interferometer. The device can scan the surface topography and extract the surface profile with its postprocessing software for calculating the surface roughness based on the ISO 25178 standard. 

Points 1, 2, 3, 4 and 6 were located at the horizontal centerline, and meanwhile, other points were located at the vertical centerline, and furthermore, 5 mm was set as the distance between each point. Electronic analytical balance (METTLER TOLEDO ME204) was used to achieve the difference in weight (before and after the processing); the material removal rate MRR can be calculated by MRR = (*Mass*_before_ − *Mass*_after_)/processing time. 

Scanning electron microscopy (SEM, ZEISS Merlin) was used to observe the microscopic surface of sapphire substrates and polishing tool. The substrate was washed within dehydrated alcohol assisted by ultrasonic cleaner for 30 min. In addition, died by high pressure gas. A piece of polishing tool was cut down and covered by the plastic wrap which was cleaned by ultrasonic cleaner and dried by high pressure gas. Before testing, the plastic wrap should be removed, the surface of substrate and polishing tool were sprayed with gold to increase electrical conductivity. Sapphire substrates were cleaned by ultrasonic cleaner for 30 min before and after polishing and dried by compressed dry gas. 

The test procedure of Shore hardness is to measure the pressed depth of the specified needle under certain conditions and then convert the depth to hardness. The indentation hardness is inversely proportional to the indentation depth and is related to the elastic modulus and viscoelasticity of the material. The LX-D type of Shore hardness tester was employed in this work. After obtaining the polishing tool, Shore hardness was tested immediately at the center of polishing tool for 5 times and the average value was used as the final Shore hardness.

## 4. Result and Discussion

### 4.1. Weight Analysis

**Figure 6:**
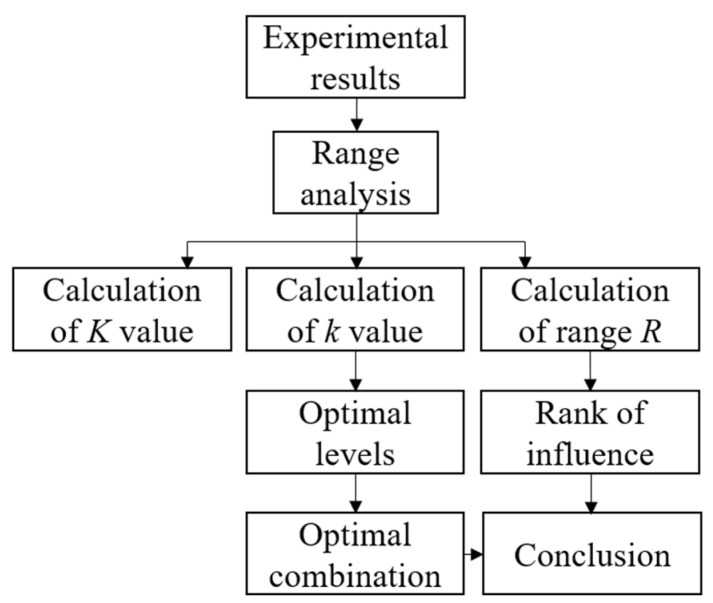
Flowchart for analyzing experimental results.

The detailed orthogonal table L_18_(3^7^) of experimental parameters for polishing sapphire and the experimental results were shown in Table 2, which was used for discussing the polishing characteristics of the proposed method in deep. The analysis process of experimental results was shown in Figure 6. The sum *K*, mean *k* and range *R* of various factors were calculated, where *K*_xy_ represents the sum of the experimental results of the *y* level (y = 1, 2, 3) under the *x*th factor (*x* = A, B, C, D, E); *k*_xy_ represents the mean value of *K*_xy_; and Range *R*_x_ represents the difference between the maximum and minimum value of *k*_x_. The initial surface roughness was around *R*_a_ 700 nm.

The order was determined by *R*_x_, higher in *R*_x_ the higher order. The optimal level was determined by the minimum *k*_x1_, *k*_x2_ and *k*_x3_ in surface roughness Ra and maximum *k*_x1_, *k*_x2_ and *k*_x3_ in material removal rate MRR.

**Figure 7:**
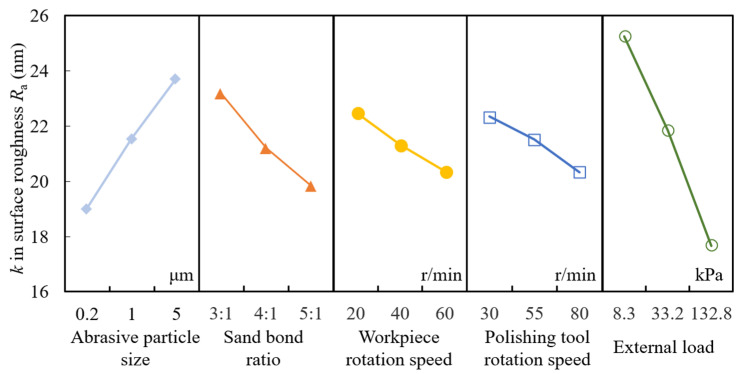
Relationship between parameters and surface roughness *R*_a_.

For intuitively explaining the change trend of the experimental results with the parameters, the relationship between parameters and surface roughness *R*_a_ was shown in Figure 7. Surface roughness can be reduced with the increase in sand bond ratio, revolution speed of workpiece and polishing tool and external load. In contrast, surface roughness increased with the increase in abrasive particle size. Smaller abrasive particle size can obtain a larger amount of particles, which result in a larger contact interface between abrasive particle and the workpiece surface and enhance solid-phase reaction intensively. In this case, material can be removed efficiently and smoother surface can be attained easily. Thus, it can be seen that the 200 nm in size achieves the smallest surface roughness compared with other sizes. The higher sand bond ratio means larger number of abrasive particles; more peak-valleys on the surface can be removed during the processing, which decreases the surface roughness in high efficiency. The sand bond ratio 5:1 performs best in smoothing the surface. The revolution speed of workpiece and polishing tool affects surface roughness less than other parameters. External load has the greatest effect on surface roughness: the greater the external load, the better the surface quality.

**Figure 8:**
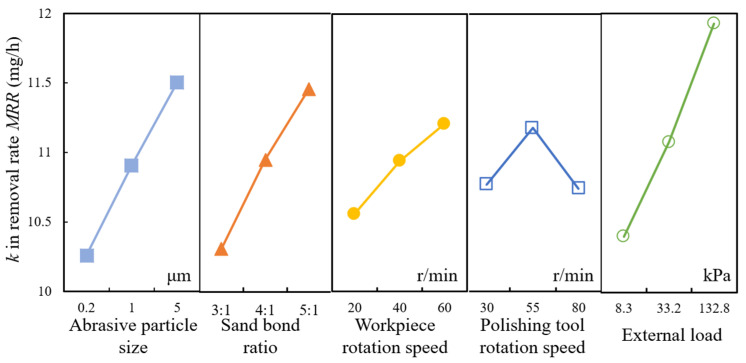
Relationship between parameters and material removal rate MRR.

The relationship between parameters and the material removal rate is shown in Figure 8. When 5 μm in abrasive particle size was employed for polishing, the average polishing force acted on each particle was larger than with a smaller size because the number of abrasive particles with 5 μm diameter was much fewer than with others. Thus, the material removal depth induced by cutting edge of abrasive particles is higher during polishing. However, larger abrasive particle size may cause overlarge polishing force on the cutting edge, which will be apt to induce micro cracks and other defects and rough surface. Higher sand bond rate improves the number of active particles, which tremendously enhance the solid phase reaction between particles and workpiece surface. Thus, the material removal rate is increased. Generally, the higher the rotation speed, the higher the material removal rate, but it is not blindly increased. Because abrasive particles need adequate time to react with workpiece surface material, the over-high speed will slow down the solid phase reaction efficiency, resulting in a decrease in the material removal rate. Larger external load can enhance the polishing force acted on single abrasive particles, which will cause a higher capacity of abrasive particles to remove material.

**Figure 9:**
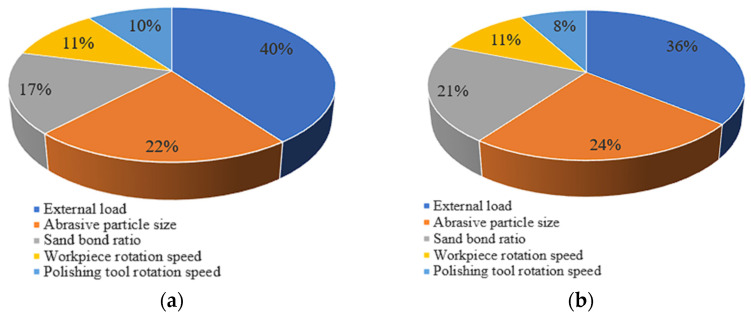
Influence percentage of each factor to surface roughness (**a**) and material removal rate MRR (**b**).

As shown in Table 3 and Table 4, R_E_ > R_A_ > R_B_ > R_C_ > R_D_, the greater the R_x_, the greater the influence of the xth factor on the index. Therefore, the influence effects are arranged in descending order: external load, abrasive particle size, sand bond ratio, revolution speed of workpiece and polishing tool. The percentage of influence is shown in Figure 9. By taking into account the surface quality, this paper proposes that the best processing conditions based on the current polishing conditions are that the particle size is 200 nm, the sand bond ratio is 5:1, the revolution speed of workpiece is 60 r/min, the revolution speed of polishing tool is 55 r/min and external load is 132.8 kPa. By taking into account the material removal rate, the best processing conditions are that the particle size is 5 μm, the sand bond ratio is 3:1, the revolution speed of workpiece is 60 r/min, the revolution speed of polishing tool is 55 r/min and external load is 132.8 kPa.

### 4.2. Polishing Tool

**Figure 10:**
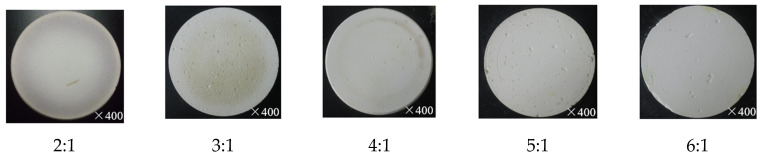
Optic pictures of polishing tool with 200 nm in abrasive particle size.

**Figure 11:**
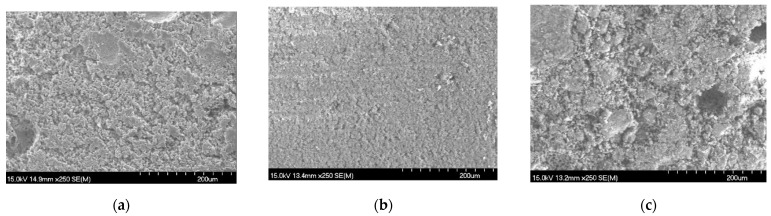
Micro view of polishing tool with different abrasive particle size. (**a**) 200 nm; (**b**) 1 μm; (**c**) 5 μm.

The polishing tool is one of the core contents of the proposed method and determines the polishing performance. In order to gain an in-depth understanding of the characteristics of the proposed method in this paper, a study on the polishing tool is carried out. The polishing tools used in this paper were made by the following process: mix–mold–wash–dry–harden–shape. The components employed in the process mainly include abrasive particles, binders, deionized water, hydrochloric acid, oxymethylene and additives (NaCl for increasing abrasive porosity, MgO and HCl for catalyst and CaO for strengthening polishing tool). As shown in Table 5, five kinds of polishing tool were obtained in this study with different compositions where deionized water, hydrochloric acid, oxymethylene and additives were constant at 1100 g, 70 g, 115 g and 179 g, respectively. The optic pictures of these obtained polishing tools with 200 nm in abrasive particle size are shown in Figure 10. With the increase in sand bonder ratio, the Shore hardness of each polishing tool decreases from 49.1 HS to 39.3 HS (49.1, 45.5, 43.4, 41.3, 39.3 HS, respectively), which means that it is difficult to mold the polishing tool when the sand bond ratio increases; in other words, the hardness of polishing tool is weakened as the decrease in concentration of bonder. Thus, once the sand bond ratio increases, the difficulty of manufacturing polishing tool increases. As shown in Figure 11, the micro views of polishing tool with different abrasive particle size (200 nm, 1 μm and 5 μm) and 5:1 of sand bond ratio were displayed to study the micro-topographies of the polishing tool. It is obvious that tight structure and smooth surface with fewer large pores is obtained when 1 μm of the particle size was used. Although the best polishing performance was obtained with 200 nm size of abrasive particle, the appearance of the attained polishing tool was not as good as others. This is because that the particle sizes in 200 nm and 5 μm are hard to disperse in the solution than particles in 1 μm size, namely, abrasive particle in 200 nm size is apt to be aggregated together in the solution because of the drastic Brownian movement and abrasive particle in 5 μm size trends to deposit in the solution due to the overlarge mass than abrasive particle in 1 μm size. Moreover, by comparing hardness with these polishing tools (particle sizes in 200 nm, 1 μm and 5 μm are 41.3 48.5, 56.6 HS, respectively), it is exhibited that a polishing tool with particle sizes in 200 nm has the lowest hardness, which is one of the main reasons for the smallest material removal rate in polishing performance. The reason is perhaps that the number of small particles is much higher than for bigger particles for a certain mass of particles; the amount of binder is not enough to bind small particles together, leading to reduction in the hardness.

**Figure 12:**
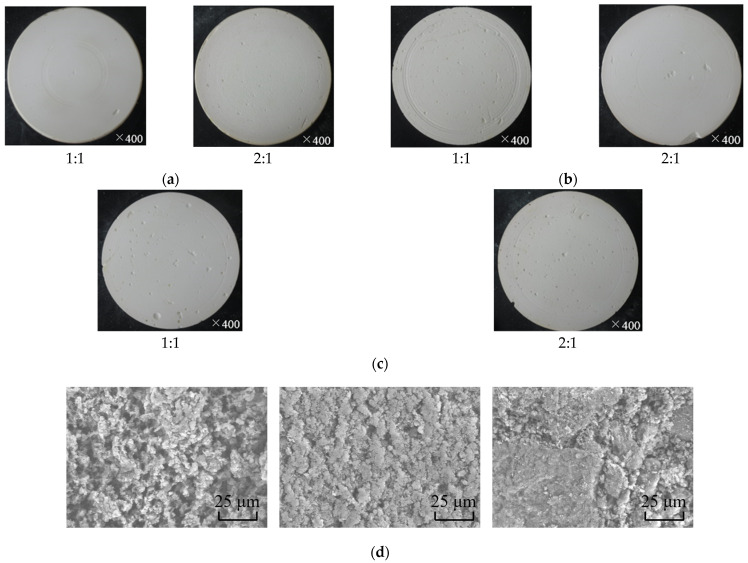
Polishing tool with mixture abrasive particles: (**a**) 200 nm and 1 μm, (**b**) 200 nm and 5 μm, (**c**) 1 μm and 5 μm and (**d**) surface topography of each polishing tool with 2:1 abrasive weight proportion.

By considering the difficulty in manufacturing polishing tool and polishing performance, the novel polishing tool, as shown in Table 6, was prepared with mixture of abrasive particle size where deionized water, hydrochloric acid, oxymethylene and additives were also constant at 1100 g, 70 g, 115 g and 179 g, respectively. The obtained polishing tool is shown in Figure 12, where (Figure 12a–d) are optic pictures of their appearance and micro topographies. These polishing tools have the hardness in 48, 46.5, 50.2, 48, 54.6 and 53.9 HS, respectively. Obviously, smoother and more intact surface of polishing tool was achieved with abrasive particles combined by 200 nm and 1 μm. However, according to Figure 12d, the polishing tool with abrasive particles combined by 200 nm and 1 μm with 2:1 abrasive weight proportion has more pores than others and has the lowest hardness, but the hardness (46.5 HS) is much higher than for the polishing tool with abrasive particles size in 200 nm (41.3 HS) and a little smaller than for the polishing tool with abrasive particles size in 1 μm (48.5 HS); the same situation can be found by comparing the hardness of a polishing tool that has abrasive particles size in 200 nm and 5 μm (2:1 abrasive weight proportion) with the polishing tool with an abrasive particles size of 5 μm, which demonstrated that the difficulty of manufacturing a polishing tool can be reduced by mixing bigger abrasive particles into small abrasive particles. 

### 4.3. Polishing Performance

**Figure 13:**
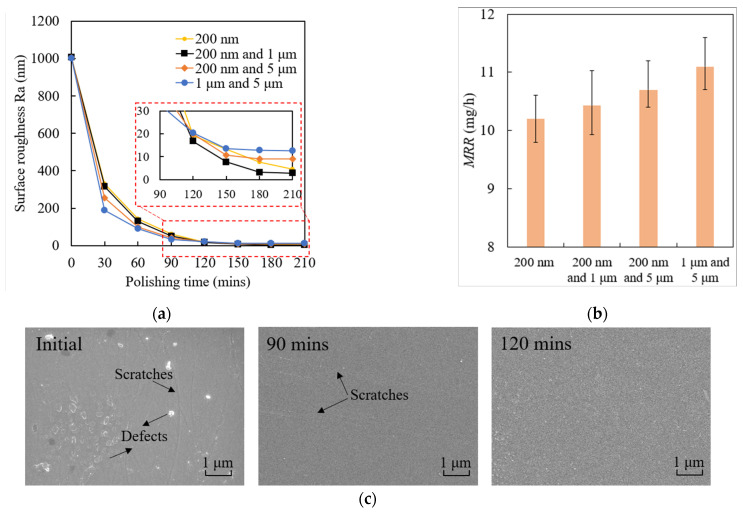
Polishing performance with mixture abrasive particles: (**a**) surface roughness *R*_a_, (**b**) material removal rate MRR, (**c**) improvement on surface morphology.

To compare the polishing performance of the mixture abrasive particles polishing tool with the single abrasive particle polishing tool, the polishing experiments were carried out. The initial surface roughness was around *R*_a_ 1000 nm. Based on the current optimized polishing conditions, the experiments were conducted under the conditions of a sand bond ratio of 5:1, a workpiece revolution speed of 60 r/min, a revolution speed of the polishing tool of 55 r/min and an external load of 132.8 kPa. The mixtures of abrasive particles of 200 nm and 1 μm, 200 nm and 5 μm, and 1 μm and 5 μm were employed in the experiments, respectively. The polishing time was 210 min. The polishing performances are displayed in Figure 13. It is obvious that each polishing tool with a mixture of abrasive particles can reduce the surface roughness *R*_a_. The polishing efficiency increases when the abrasive particle changes in size from 200 nm to 1 μm and 5 μm in the first 60 min. After that, the polishing efficiency starts to be reduced, except for the abrasive particle size of 200 nm. The best surface roughness of *R*_a_ 2.8 nm was obtained by using a mixture abrasive particles with 200 nm and 1 μm, rather than abrasive particles with 200 nm (surface roughness *R*_a_ 4.5 nm). Although it is possible that better surface roughness can be obtained by extending the polishing time, it was obvious that the mixture of 200 nm and 1 μm abrasive particles performed better within 210 min polishing. Therefore, by considering the difficulty in using the molding polishing tool and the polishing performance, a polishing tool with a 200 nm and 1 μm mixture of abrasive particles is recommended for the first 210 min polishing. As for the material removal rate, MRR increases when abrasive particle changes in size from 200 nm to 1 μm and 5 μm. This phenomenon was coincident with the general polishing rule that larger abrasive particles used in polishing process perform at a higher material removal rate. Larger abrasive particles were fewer in number within the polishing tool compared with smaller abrasive particles in these experiments, which results in each larger abrasive particle suffering from a higher polishing force compared to smaller abrasive particles. Thus, sizes of 1 μm and 5 μm induce the highest material removal rate. The improvement in surface morphology with the variation in polishing time by using the 200 nm and 1 μm mixture of abrasive particles is displayed in Figure 13c. The initial surface was rough with micro-bulges and micro-holes which were induced by the pre-process. Obviously, the surface morphology changes tremendously in the first 90 min of polishing and slows down for the rest of the time. This is because the peaks and valleys were removed abundantly at the beginning of polishing; the distances of peaks to valleys were reduced quickly with the increase in polishing time, which contribute to decreasing surface roughness rapidly. However, the reduction rate of the distances of peaks to valleys was reduced continuously during polishing process. After 90 min of polishing, these defects were gradually diminished and only few micro scratches were found on the surface. At the end of polishing process, the surface was smoothed without any distinct defects. 

## 5. Conclusions

To further develop the application of the semi-fixed abrasive grains polishing technique, the polishing characteristics of the proposed polishing method was investigated by experimental analysis; consequently, the following conclusions are drawn:

(1) External load affects the surface roughness and material removal rate the most, then the abrasive particle size, sand bond ratio, revolution speed of workpiece and polishing tool. The optimized polishing conditions are when the particle size is 200 nm, the sand bond ratio is 5:1, the revolution speed of workpiece is 60 r/min, the revolution speed of polishing tool is 55 r/min and external load is 132.8 kPa, when surface quality was required. However, once the material removal efficiency was taken in account, the appropriate polishing conditions were changed to 5 μm in particle size, 3:1 in sand bond ratio, 60 r/min and 55 r/min in the revolution speed of workpiece and polishing tool and 132.8 kPa in external load.
(2) Because the difficulty in molding polishing tool increases with the decrease in particle size, although polishing tool with 200 nm particle size has the best performance on polishing, it was difficult to obtain such a polishing tool. Therefore, a polishing tool with a mixture of particles is proposed. The results demonstrated that the difficulty of manufacturing such a polishing tool can be reduced by mixing bigger abrasive particles into small abrasive particles.
(3) The polishing performance was investigated with different polishing tools containing different abrasive particle sizes. The results show that the polishing tool with 200 nm and 1 μm particle size performs best in the first 210 min of polishing. However, the polishing tool with 200 nm has the tendency to obtain better surface roughness by extending the polishing time.

## Figures and Tables

**Table 1:** Designed orthogonal table.

Level	Factors						
	A (μm)	B	C (r/min)	D (r/min)	E (kPa)	F	G
1	0.2	3:1	20	30	8.3		
2	1	4:1	40	55	33.2		
3	5	5:1	60	80	132.8		

**Table 2:** Experimental results (*R*_a_ and MRR).

Expno.	Parameters							Results	
	A	B	C	D	E	F	G	*R*_a_ (nm)	MRR (mg/h)
1	1	1	1	1	1	1	1	26.5	8.3
2	1	2	2	2	2	2	2	18.3	10.4
3	1	3	3	3	3	3	3	10.2	11.9
4	2	1	1	2	2	3	3	23.4	10.1
5	2	2	2	3	3	1	1	15.3	11.7
6	2	3	3	1	1	2	2	22.6	10.5
7	3	1	2	1	3	2	3	20.9	11.9
8	3	2	3	2	1	3	1	25.7	11.2
9	3	3	1	3	2	1	2	21.5	11.7
10	1	1	3	3	2	2	1	19.4	9.7
11	1	2	1	1	3	3	2	17.6	10.9
12	1	3	2	2	1	1	3	22.4	10.3
13	2	1	2	3	1	3	2	27.3	9.5
14	2	2	3	1	2	1	3	22.6	11.3
15	2	3	1	2	3	2	1	17.8	12.3
16	3	1	3	2	3	1	2	21.5	12.5
17	3	2	1	3	1	2	3	28.2	10.1
18	3	3	2	1	2	3	1	23.6	11.8

**Table 3:** Intuitive analysis table for surface roughness *R*_a_.

Item	Surface Roughness *R*_a_ (nm)				
	A	B	C	D	E
*K*_x1_	114.4	139.0	135.0	133.8	152.7
*K*_x2_	129	127.7	127.8	129.1	128.8
*K*_x3_	141.4	118.1	122.0	121.9	103.3
*k*_x1_	19.067	23.167	22.500	22.300	25.450
*k*_x2_	21.500	21.283	21.300	21.517	21.467
*k*_x3_	23.567	19.683	20.333	20.317	17.217
*R*_x_	4.500	3.484	2.167	1.983	8.233
Order	E > A > B > C > D				
Optimal level	A_1_ B_3_ C_3_ D_3_ E_3_				
Optimal combination	A_1_B_3_C_3_D_3_E_3_				

**Table 4:** Intuitive analysis table for material removal rate MRR.

Item	Material Removal Rate MRR (mg/h)				
	A	B	C	D	E
*K*_x1_	61.5	62.0	63.4	64.7	59.9
*K*_x2_	65.4	65.6	65.6	66.8	65.0
*K*_x3_	69.2	68.5	67.1	64.6	71.2
*k*_x1_	10.250	10.333	10.567	10.783	9.983
*k*_x2_	10.900	10.933	10.933	11.133	10.833
*k*_x3_	11.533	11.417	11.183	10.767	11.867
*R*_x_	1.283	1.084	0.616	0.366	1.884
Order	E > A > B > C > D				
Optimal level	A_3_ B_3_ C_3_ D_2_ E_3_				

**Table 5:** Compositions of polishing tool with 200 nm abrasive particles.

	Sand Bond Ratio	Abrasive Particle (g)	Bonder (g)
No.1	2:1	800	400
No.2	3:1		266.7
No.3	4:1		200
No.4	5:1		160
No.5	6:1		133.3

**Table 6:** Compositions of polishing tool with mixture abrasive particles.

	Abrasive Weight Proportion	Sand Bond Ratio	Abrasive (g)	Bonder (g)
200 nm + 1 μm	1:1	5:1	800	160
	2:1			
200 nm + 5 μm	1:1			
	2:1			
1 μm + 5 μm	1:1			
	2:1			

## Abbreviations

Magnetorheological finishing	MRF
Magnetic compound fluid	MCF
Chemical mechanical polishing	CMP
Material removal rate	MRR
Scanning electron microscopy	SEM
